# A typology of nurses' interaction with relatives in emergency situations

**DOI:** 10.1177/09697330221128902

**Published:** 2022-10-31

**Authors:** Nadia Primc, Sven Schwabe, Juliane Poeck, Andreas Günther, Martina Hasseler, Giovanni Rubeis

**Affiliations:** 1 Institute of History and Ethics of Medicine, 9144 University of Heidelberg, Heidelberg, Germany; 2 Institute for General Practice and Palliative Care, 9177 Hannover Medical School, Hanover, Germany; 3 Institute of General Practice, 39065 University Hospital Jena, Jena, Germany; 4 Fire Department, 524823 City of Braunschweig, Braunschweig Germany; 5 Faculty of Health Sciences, 120199 Ostfalia University of Applied Sciences, Wolfenbüttel Germany; 6 Division Biomedical and Public Health Ethics, 467773 Karl Landsteiner University of Health Sciences, Krems an der Donau, Austria

**Keywords:** Nursing home, emergency, relatives, ethics, avoidable hospital transfers

## Abstract

**Background:**

In nursing homes, residents’ relatives represent important sources of support for nurses. However, in the heightened stress of emergency situations, interaction between nurses and relatives can raise ethical challenges.

**Research objectives:**

The present analysis aimed at elaborating a typology of nurses’ experience of ethical support and challenges in their interaction with relatives in emergency situations.

**Research design:**

Thirty-three semi-structured interviews and six focus groups were conducted with nurses from different nursing homes in Germany. Data were analysed according to Mayring’s method of qualitative content analysis.

**Participants and research context:**

Participants were licensed nurses working in nursing homes.

**Ethical considerations:**

Ethical approval was granted by Ostfalia University of Applied Sciences (02.07.2020) and the Ethics Committee of Hannover Medical School (Nr. 8866_BO_K_2020; 27.01.2020). Interviewees were anonymised and focus group were pseudonymised during transcription. All participants provided written consent.

**Findings/results:**

In emergency situations, relatives can represent important sources of support for nurses. However, they may also give rise to different challenges, relating to four ethical conflicts: (1) the challenge of meeting the information needs of relatives while providing appropriate care to all residents; (2) the challenge of managing relatives’ demands for hospitalisation when hospitalisation is not deemed necessary by nurses; (3) the challenge of managing relatives’ demands for lifesaving treatment when such treatment contradicts the will of the resident; and (4) the challenge of attempting to initiate hospitalisation when relatives oppose this course of action. Several external factors make these conflicts especially challenging for nurses: fear of legal consequences, a low staffing ratio, and a lack of qualified nursing staff.

**Conclusions:**

Conflict between nurses and relatives typically revolves around hospitalisation and the initiation of lifesaving treatment. Whether nurses perceive interaction with relatives as supportive or conflictual essentially depends on the quality of the relationship, which may be negatively influenced by a number of external factors.

## Background

Emergency situations are particularly challenging for nurses in nursing homes. As the majority of nursing homes do not have doctors such as general practitioners (GPs) on site, nurses are usually the first to respond to emergency situations.^1^ Emergency situations are characterised by physical, behavioural, or mental changes in residents, for which immediate medical care is deemed necessary by either the nurses, the residents, or third parties (e.g. relatives, doctors).^2^ While some emergency situations can be independently handled by nurses, in other situations, nurses must call on other health care professionals (e.g. doctors, emergency medical service (EMS)) for assistance.

Due to the life-threatening nature of emergency situations, nurses must make timely decisions, while coordinating and communicating with people inside and outside the nursing home (e.g. residents, doctors, EMS).^3^ In these situations, residents’ relatives represent important contact persons and pillars of support for nurses.^4^ However, nursing staff may also feel pressured by relatives.^5^ Thus, they may perceive interaction with relatives as ethically challenging, forcing them to balance different responsibilities towards the residents, the relatives and possible other stakeholders (i.e. nursing home management staff, nursing facilities, doctors, EMS).

Some research has explored family-centred care and the role of relatives in determining nursing care in nursing homes.^6^ In particular, studies have focused on disagreement between relatives and nursing home staff concerning life-prolonging treatment and hospitalisation^7–9^; the involvement of relatives in dementia care^10^; ethical conflicts concerning everyday care^8^; relatives’ perceptions of care quality, aspects of dignity, and coercive measures^11–14^; ethical challenges related to next of kin^15^; and relatives’ role in promoting residents’ autonomy.^16–17^ Further studies have explored the role of relatives in decision-making and the prevention of hospitalisation.^18–19^ In emergency situations, nurses may experience relatives as active supporters or troublemakers, or something in between.^4^ To the best of our knowledge, no study has produced an in-depth analysis of the ethical challenges faced by nurses in their interaction with relatives in emergency situations, or the kinds of support that relatives may offer nurses in their effort to provide appropriate care and to make ethically reflected decisions in emergency situations. The present study aimed at delineating the ethical dimension of the roles of relatives in emergency situations,^4^ and thereby laying the foundation for further research on ethically reflective interaction between nurses and relatives in emergency situations.

## Methods

### Research objective

The present analysis aimed at elaborating a typology of nurses’ experience of ethical support and challenges in their interaction with relatives of nursing home residents in emergency situations. Given the diversity of approaches in the field of nursing ethics, the normative analysis assumed four fundamental commitments as central to the interaction and decision-making of nurses in emergency situations: (1) to respect the will and autonomy of residents; (2) to provide the best possible care (according to recognised standards of care); (3) to treat relatives of residents with respect; and (4) to reduce, as much as possible, the stress and strain on relatives that is commonly associated with emergency situations. In some situations, nurses encounter conflict between these commitments. Thus, the present study assumed a hierarchy of these obligations in alignment with the legal framework in Germany, with the will and welfare of residents given highest priority. The qualitative data generated a more detailed account of the ethical obligations and conflicts that nurses perceived in emergency situations. The methodical approach was based on the COREQ checklist for reporting qualitative research.^20^

### Research design and participants

Thirty-three semi-structured individual interviews (September 2020–October 2020) and six focus groups (September 2020–April 2021) were conducted with nursing staff from different nursing homes in Germany, as part of a larger interdisciplinary research project (acronym ‘NOVELLE’) that aims at developing and implementing guidelines for emergency management in nursing homes. All 33 interviews were conducted by two authors (NP, female; GR, male), and each lasted approximately 45–75 min (average duration: 55 min). Both authors have a background in philosophy, long-standing experience in the field of medical ethics and training in qualitative methods. All interviews were conducted in person in nursing homes, with the authors alternating for each interview. Both authors were present at all interviews, and the interview style and topics were discussed intermittently between them. The focus groups were moderated by two authors (JP, male psychologist; SS, female sociologist), and each lasted 92–122 min (average duration: 107 min). Both authors have long-standing experience with qualitative methods and group discussions. Due to COVID-19 restrictions, the focus groups were conducted via a video conferencing tool.

While the interviews focused on ethical aspects of emergency management in nursing homes (prior to the development of emergency management guidelines), the focus groups aimed at developing guidelines for emergency management. Interview and focus group guides were developed on the basis of prior research and exchange within the larger research project. Subsequently, the guides were tested and discussed by the research team, which included experts from the fields of nursing science, emergency medicine, general medicine, medical ethics, sociology and psychology. The interview guide consisted of more than 40 questions, covering 10 general topics (see Table 1). Table 2 presents the discussion guide for the focus groups.

**Table 1.** Interview guide topics (based on Schwabe et al., 2022^4^).

1	Personal professional experience and areas of responsibility
2	Definitions and interpretation of emergency situations
3	Decision-making and the use of existing guidelines in emergency situations
4	Cooperation with other professional groups in emergency situations
5	Challenges in dealing with emergency situations
6	Need for support in emergency situations
7	Consideration of residents’ personal will and desires in emergency situations
8	General framework for good nursing care in emergency situations
9	Possibilities for implementing guidelines in emergency situations
10	Complementary factors in dealing with emergency situations

**Table 2.** Discussion guide for the focus groups (based on Schwabe et al., 2022^4^).

1	Please tell us about your experiences of emergency situations in your nursing home, such as … [a resident fell, a resident’s vital signs were derailed, …]
2	Now, we would like to focus on single phases and challenges of emergency management
2.1	How do nurses’ levels of experience, safe practices and qualifications affect emergency management?
2.2	How does staffing affect emergency management?
2.3	How does the accessibility of GPs and medical on-call services affect emergency management?
2.4	What role do relatives play in emergency management?
2.5	What role does the resident play in emergency management? What happens if the resident’s will is not known?
2.6	What other challenges do you face in emergency management?
3	How do you handle the challenges of emergency management?
3.1	How do you deal with insecure nursing staff?
3.2	How do you deal with insufficient staffing?
3.3	How do you deal with the poor accessibility of GPs and medical on-call services?
3.4	How do you deal with challenging relatives?
3.5	How do you cope when you don’t know the resident’s will?
3.6	How do you deal with [other challenges, see question 2.6]?

Interviewees were licensed nurses (i.e. nurses who had undergone a 3-years vocational training programme) who were currently working in nursing homes in Germany. Purposive sampling was used, and participation was voluntary. The inclusion criteria for the interviewees were nurses who were: (a) registered and (b) working for one of the nursing homes enrolled in the larger research project. Thus, all nursing homes that were enrolled in the larger research project were contacted via telephone, email or in person and asked to nominate licensed nurses who might be willing to participate in interviews or focus groups. The goal was to recruit at least two nurses from each nursing home for an interview, and this was achieved for all but one nursing home, which cancelled the interviews at the last minute. The inclusion criteria for the focus group participants were nurses or doctors who were (a) registered and (b) had experience managing emergency situations in nursing homes. The final sample of focus group participants consisted mainly of licensed nurses, though two focus groups included other experts (e.g. doctors, medical ethicists). Each focus group included three to five participants. Some nurses participated in more than one focus group, resulting in a total of 19 nurses in the sample of focus group attendees. Participants were free to decide whether to join only one or several focus groups, as each focus group dealt with a different emergency situation or topic (i.e. a fall, derailed vital signs, further emergency management, assessment of an emergency situation, pain, breathlessness). Data saturation was achieved, as, in the final interviews and focus group, the contents were repeated and no new challenges or courses of action in dealing with emergency situations were raised. All interviews and focus groups were conducted in German.

### Data analysis

Interviews and focus groups were recorded, transcribed verbatim and analysed using qualitative content analysis, according to Mayring.^21^ Content analysis was used to abstract and describe nurses’ conceptualisation of ethical responsibilities and challenges in their interaction with relatives in emergency situations. The MAXQDA® 2020 software was used to analyse transcripts. In the first step, interview and focus group transcripts were analysed separately by the responsible researchers (i.e. NP and GR for the interviews; JP and SS for the focus groups). Comparison of the coding results showed that interaction with relatives was a relevant subtheme in both groups of transcripts. In the second step, all subcodes referring to nurses’ interaction with relatives were analysed and re-coded by two researchers (NP, GR), focusing on: (a) different forms of support that relatives offer to nurses in their effort to provide appropriate care to residents in emergency situations, and (b) ethical challenges that nurses encounter in their interaction with relatives in emergency situations. Inter-coder reliability was obtained through regular discussion of the codes and coding results, with any discrepancies discussed until consent was achieved. The present study represents a secondary analysis of the qualitative data,^22^ since the primary study did not explicitly target the ethical aspects of nurses’ interaction with relatives, but more generally factors that may have a positive or negative influence on decision-making in emergency situations.

During the re-coding process, several inductive categories and subcategories emerged with respect to the types of support and ethical conflict that nurses perceived in their interaction with relatives. A typology of these supportive and challenging factors was established to systematise inductive categories and subcategories according to the assumed ethical commitments of nurses (e.g. respecting the will of relatives, providing appropriate care to all residents, supporting and informing relatives).

## Ethical considerations

Ethical approval was granted by the Ostfalia University of Applied Sciences (02.07.2020) and the Ethics Committee of Hannover Medical School (Nr. 8866_BO_K_2020; 27.01.2020). Interviews were anonymised, group discussions were pseudonymised during transcription, and audio recordings were deleted following transcription. All information that could potentially identify the participating nursing homes (e.g. number of beds) was deleted or changed in the transcripts of interviews. Additionally, interviews were numbered consecutively and all personal data were deleted. Since some nurses participated in more than one focus group, pseudonymisation was used. All interview and focus group participants were informed about the study aims and provided written consent.

## Results: Typology of supportive and challenging factors in nurses’ interaction with relatives

Table 3 provides an overview of the established typology.

**Table 3.** Typology of supportive and challenging factors in nurses’ interaction with relatives.

Support from relatives in emergency situations	Ethical conflicts with relatives in emergency situations
• Procuring medicine from the pharmacy	• Meeting relatives’ information needs while providing appropriate care to all residents
• Taking residents to the doctor’s office	• Managing relatives’ demand for hospitalisation that is not deemed necessary by nurses
• Accompanying residents during hospital transfers	• Managing relatives’ demand for treatment that contradicts the will of the resident
• Supporting nursing staff in their efforts to communicate with GPs and residents	• Managing relatives’ opposition to hospitalisation that is deemed necessary by nurses
**Overarching factors with a negative impact on nurses’ interaction with relatives:**	
Fear of legal consequences from relatives	
Low staff-to-resident ratio	
Lack of qualified nursing staff	

## Support from relatives in emergency situations

The nurses stressed that communication with relatives was an important aspect of their work, and that relatives could represent important sources of support in emergency situations.

‘That also takes a lot of work off your hands in terms of care. So we involve them [the relatives] a lot here. Also, with doctor’s visits and so on.’ (Interview 4)

The support provided by relatives could take various forms, including procuring medicines from the pharmacy, taking residents to the doctor’s office, accompanying residents during hospital transfers and supporting nursing staff in their efforts to communicate with GPs and residents. In some cases, nurses called on relatives to pressure GPs to respond more quickly, or to convince residents that hospitalisation was in their best interest.

‘Sometimes it makes sense if the relatives put pressure on the GP as well, because then he calls back faster [...]’ (Focus group 5)

In one interview, a nurse recalled a situation in which a resident initially refused hospitalisation, even though this was highly recommended by the nurse and the consulted doctor. Because of the resident’s refusal, the nurse contacted a former neighbour of the resident and asked her to come to the nursing home to speak to the resident. As a result of the neighbour’s coaxing, the resident finally agreed to hospitalisation. The nurse described this episode as follows:

‘But I didn't let it go. I called the neighbour, she came here and now she [the resident] is in the hospital. So that was this form of persuasion. Yes, these are situations in which I think you can still do something.’ (Interview 13)

In all these cases, the nurses saw relatives as supportive of their efforts to ensure the best care for all residents.

## Ethical conflicts with relatives in emergency situations

Conversely, the nurses described some interactions with relatives in emergency situations as burdensome.

### Meeting relatives’ information needs while providing appropriate care to all residents

The nurses described that emergency situations required a high degree of coordination. Specifically, in these situations, nurses had to assess the emergency situation and make decisions regarding emergency care. They also had to contact external actors (e.g. doctors, EMS) and prepare documents for hospital referral, while managing the emergency situation and caring for other residents. The nurses described that, amidst these demands, the work of contacting and talking to relatives represented an additional burden, particularly because relatives were usually highly concerned about the resident’s health. Thus, in emergency situations, the nurses were confronted with a conflict between their duty to ensure appropriate care for all residents and their need to meet the urgent information needs of relatives.

‘We had a patient here, very recently, who had a stroke. And her daughter was upset that we didn’t call her first instead of the ambulance. […] when someone has a stroke or a heart attack, I don’t call the relatives first and ask whether we should send them to hospital [*laughs slightly*], but of course I send them immediately away, right?’ (Interview 11)

The nurses elaborated that relatives also exerted pressure on nursing home managers, to whom they frequently addressed complaints about what they saw as the inappropriate involvement of residents’ family members in emergency situations. Some nurses criticised nursing home managers for their failure to provide sufficient support:

‘But unfortunately, it is also the case that the nursing homes are now [*short pause*], the management is no longer as rigorous as it used to be, you know? In the past, relatives were told: “Well, if you don’t like it here at all and if you are completely dissatisfied, then change the home.” They don’t do that anymore. They rather try to please the relatives in case of doubt. So that no one gets in any trouble. And then it’s more the resident who falls by the wayside than the relative.’ (Interview 16)

### Managing relatives’ demand for hospitalisation that is not deemed necessary by nurses

The nurses described that most ethical challenges arose when relatives were involved in decision-making in emergency situations. In many cases, the conflict referred to previous arrangements (e.g. general advance directives; written directives pertaining specifically to emergency situations; verbal agreements with residents, relatives and doctors). For instance, an often-mentioned conflict pertained to emergency situations in which relatives demanded hospitalisation or an immediate visit to the doctor, although this was not deemed necessary by nurses (e.g. after a fall or minor circulatory derangement):

‘[…] the relatives usually tip the scale, so that an avoidable hospitalisation cannot be avoided in that case.’ (Focus group 1)

The nurses claimed that they were well aware that avoidable ambulance calls and hospital admissions placed a burden on other health care professionals and the health system, in general. They also stressed that it was in the resident’s interest to avoid unnecessary hospital admissions. However, due to pressure from relatives, they sometimes found themselves compelled to act against their professional judgment and ethical principles. The nurses described that this conflict was even more acute during the COVID-19 pandemic, when hospitalisation was associated with an increased risk of infection with SARS-CoV-2 and, due to restrictions, residents could not be accompanied by their relatives. The nurses reported that their professional assessment was often not taken seriously by relatives. As there was usually no on-site doctor at the nursing homes, pressure from relatives could lead to unnecessary hospital admissions:

‘[…] because in the view of the relatives, we are not the doctor. And for many, the doctor is still the one who has the say, and then it would be desirable if they came here, but of course they can’t always, you can’t expect them to be here immediately every time a resident falls without injuries, that’s simply not possible.’ (Focus group 1)

The nurses described that they would appreciate closer cooperation with GPs, who, in their opinion, did not always listen to their professional judgement:

‘[…] they should get in touch with us directly and ask for our assessment, or not just let the relatives tell them everything and believe them, but rather ask us what is really the case, because I think it is often the case that the relatives completely overdramatise when telling the GP what happened and they have not even heard our professional opinion. And I believe that more support would complement this in such a situation, when they really ask us for our opinion.’ (Focus group 1)

### Managing relatives’ demand for treatment that contradicts the will of the resident

Another type of conflict pertained to situations in which relatives demanded the initiation of lifesaving treatment, even though the nurses were convinced that such treatment was contrary to the verbally stated, documented or presumed will of the resident, or even prior agreements with the resident, relatives and GPs.

‘And of course, it’s not so easy to find a compromise between the resident’s wishes and the wishes of the relatives and the medical necessity of the whole thing, right? And that is difficult, isn’t it? That is, and often, uh, relatives demand that you, uh, do something, that means call the ambulance, you know? And that’s just the way it is, isn’t it? And then you get also completely insulted if you don’t do that.’ (Interview 12)

‘Um, that’s difficult, because such people sometimes take legal action. Which then hangs round my neck, where I know that everything will be picked apart, that someone will investigate it and you name it. And that’s also something that puts you under pressure.’ (Interview 10)

The nurses described that, in emergency situations, incorporating the resident’s will could be challenging in itself, especially when the situation was unexpected. In unexpected situations, nurses needed to obtain relevant information concerning the resident’s wishes, so they could make immediate decisions. If relatives then questioned the documented or alleged will (or arrangements made between nurses, residents, relatives and GPs), nurses could feel additionally burdened. Above all, nurses feared legal reprisal from relatives in such situations.

‘Um, well, you already learn during your training that you always are one step away from jail [*laughs*]. But that’s how it is.’ (Interview 10)

### Managing relatives’ opposition of hospitalisation deemed necessary by nurses

Another type of conflict arose when nurses wanted to initiate hospitalisation but relatives opposed this line of action. From an ethical perspective, this type of conflict differs from that described in the preceding subsection: the abovementioned conflict involves nurses’ efforts to reconcile and uphold the will of residents and the wishes of relatives; in contrast, the conflict described in this subsection pertains to nurses’ obligation to provide the best possible care to residents according to recognised standards of care, while managing the desires of relatives. The nurses described that, in some emergency situations, they felt pressured to act against their professional judgment and ethical commitment to delivering care according to recognised standards of care:

‘And um, there are still sometimes relatives who assess situations differently than the residents themselves or the person in an emergency himself and who then always says: “That doesn’t have to be. No, I don’t want that for my mother or father.” And yes, that is always difficult.’ (Interview 6)

## Overarching factors with a negative impact on nurses’ interactions with relatives

Alongside the fear of legal consequences, a number of additional factors were identified that made dealing with the abovementioned conflicts even more challenging for nurses. In particular, the nurses identified low staffing ratios and a lack of qualified nursing staff in Germany. They also reported that high workloads made it more difficult for them to appropriately deal with conflicts with relatives. The nurses described that, in nursing homes with a shortage of qualified staff, they were often forced to fill staffing gaps in other wards, in which they did not know the residents and their relatives. Furthermore, many nursing homes had to rely on temporary workers who, according to the nurses, did not know the residents well and were unaware of existing arrangements between primary carers, residents, relatives and GPs. The nurses described that close relationships with residents and relatives enabled them to make ethical decisions in emergency situations. When asked about possible conflict with relatives in emergency situations, one nurse replied:

‘If you explain it well to them and explain it from the perspective of the resident, of the person being cared for, if you explain it relatively well to them, then they will come along. […] What can lead to problems is, for example, if you don’t work in your ward, in your residential area. Instead, you have a change of ward because a nurse is missing. As it always is [*laughs lightly*]. That was a great [*sarcastic tone*] situation that also happened to me. It had already been clarified, no more hospital admission, no more ambulance, nothing more. I didn’t have that information. That was a nightmare, of course. But that’s just the way it is, isn’t it?’ (Interview 22)

## Discussion

The qualitative analysis showed that the nurses were confronted with conflicting ethical commitments in emergency situations. These conflicts concerned, above all else, tension between the nurses’ ethical obligations towards residents (i.e. respecting the will of residents and preventing harm to their physical and mental well-being) and the demands placed on them by relatives. The results highlighted that, in emergency situations, the nurses made decisions and prioritised tasks and obligations – not only to care for the resident in need of emergency care, but also to maintain care for all residents. Several studies have suggested that a high workload and low staffing ratio in nursing homes may negatively impact the quality of nursing care,^23^ through the implicit rationing of nursing care (i.e. ‘care left undone’, ‘unfinished care’, ‘missed care’). The implicit rationing of nursing care signifies that, due to a lack of time (or other resources), nurses are regularly unable to perform all nursing tasks considered necessary^24^ to meet professional and ethical standards of care. Furthermore, EMS paramedics in Germany have recognised that high workloads, low staffing ratios and a shortage of qualified nurses may contribute to preventable hospital transfers.^25^

At the same time, the nurses in the present study felt obliged to meet the needs of residents’ relatives, claiming that good relationships and communication with relatives were important for maintaining ethical and high-quality care in emergency situations. Similar views have been reported for the care of nursing home residents, in general.^7^ The results of the present analysis suggest that staff shortage and low staffing ratios may negatively affect nurses’ relationships with relatives. As a consequence, previous agreements and living wills may stand at risk of being ignored, especially in emergency situations that are unanticipated (e.g. emergencies that occur prior to the initiation of end-of-life and palliative care). In the present study, nurses admitted that a fear of legal reprisal sometimes led to residents' wills being ignored due to pressure from relatives, who themselves experienced emergency situations as highly emotionally and morally stressful. These factors may contribute to explaining why advance care planning (ACP) does not always seem to have the desired effect.^26^

The present analysis identified four types of conflicts experienced by nurses: (1) conflict due to the difficulty involved in balancing relatives’ urgent need for information with nurses’ obligation to ensure appropriate care for all residents; (2) conflict due to relatives’ demand for hospitalisation that is not deemed necessary by nurses; (3) conflict due to relatives’ demand for the initiation of lifesaving treatment that apparently contradicts residents’ wishes; and (4) conflict due to relatives’ disagreement with nurses’ professional recommendation to initiate hospitalisation. Given the uniqueness of each emergency situation, no generally applicable recommendations can be provided for the ethical management of these conflicts. However, some essential ethical aspects may be highlighted on the basis of the normative framework used in the present analysis (see the ‘Methods’ subsection, above) and the legal framework in Germany.

In the event of the first type of conflict (i.e. with relatives’ information needs conflicting with nurses’ obligations of care), we suggest that nurses first and foremost attend to the well-being of residents. Of course, relatives have a right to be informed and nurses should be committed to treating them with respect, and to reducing, as much as possible, the stress and strain on relatives that is commonly associated with emergency situations. However, the health, safety and autonomy of residents must be considered the higher ethical (and legal) good.

The second type of conflict refers to unnecessary hospital transfers (i.e. situations in which hospitalisation is deemed inappropriate by nurses according to current standards of care, but relatives insist on it anyway). Unnecessary hospital transfers pose potential risks for elderly residents, pertaining to a general deterioration of health, iatrogenic events, medication errors, delirium and the interruption of nursing care.^3,15^ More specifically, research has shown that nursing home residents show an increased risk of morbidity and mortality after hospital admission.^4^ Given this evidence, nurses should avoid any hospital transfer that they deem unnecessary, especially amidst the current COVID-19 pandemic. Thus, in this type of conflict, the health, safety and autonomy of residents should again be considered the higher good.

In the event of the third type of conflict (i.e. in which relatives demand lifesaving treatment despite the stated/documented/alleged will of the resident), the will of the resident must be prioritised over the concerns of relatives, despite relatives’ quite understandable fear of losing a loved one. As most nurses in the present study claimed to give the resident’s will the highest importance, it seems the fear of legal reprisal from relatives was most likely to tempt them (and sometimes the doctors they called on) to give in to relatives’ pressure. However, a recommended alternative pathway would be to provide relatives with emotional and psychological support, while maintaining the will of the resident.

Concerning the fourth type of conflict (i.e. wherein nurses highly recommend hospitalisation but the relatives oppose this), nurses should act in the best interest of the resident’s well-being. If mentally competent, residents have a moral (and also legal, in Germany) right to make seemingly unreasonable decisions on their own behalf (i.e. to refuse treatment or hospitalisation even if this is regarded as their best treatment option). However, this right to make unreasonable decisions is not extended to relatives, who, in emergency situations, should instead advocate for the resident’s will.^27^

The present study comprised part of an ongoing research project that aims at tackling the abovementioned challenges through the development of guidelines for emergency management in nursing homes. The results of the ongoing project will show the extent to which these guidelines may help to resolve any ethical conflicts that arise in emergency situations. Further research is needed to uncover the possible role played by clinical ethics counselling in the management of conflict in emergency situations in nursing homes. However, a specific challenge of emergency situations in nursing homes is the time pressure, which makes ethical counselling difficult to implement. Instead, regular discussion with residents, relatives and doctors, as well as written agreements (e.g. as part of ACP) are generally recommended to prevent conflict in end-of-life decision-making.^26^ In the present study, the nurses agreed that such discussions and agreements would be valuable in preventing conflict and unnecessary hospital transfers in emergency situations. Nevertheless, they also noted the limitations of this approach: it can only produce benefits under adequate conditions (i.e. in nursing homes with adequate staffing and primary nursing care models).

Whether the nurses perceived interaction with relatives as supportive or conflictual essentially depended on the quality of their relationships with relatives,^4^ which could be negatively influenced by a number of external factors (i.e. high workloads, low staffing ratios, a shortage of qualified nursing staff, a general lack of recognition of the professional expertise of nurses). Some nurses also reported a lack of support from nursing home managers. Thus, focus must be given to these contextual factors, in order to improve interaction with relatives and quality of care in emergency situations.

## Strengths and limitations of the study

The present study was based on a secondary analysis of qualitative data.^22^ Thus, further research is needed to explicitly address the ethical challenges that arise in nurses’ interaction with relatives in emergency situations. Although the interviews and focus groups were planned and analysed independently, ethical conflict with relatives emerged as a subcode in both groups of transcripts. All 33 interviewees addressed the role of family members at least once, and the six focus groups produced 44 codings about relatives. These data were considered valid for addressing the research question, as the ethical challenges that arise in nurses’ interaction with relatives are linked to the original study topic of the management of emergency situations in nursing homes.

Another limitation of the study is that it only included the views of nurses (and not, e.g. relatives, GPs or EMS). For this reason, it was not possible to determine the reliability and adequacy of nurses’ judgments, especially where these seemed to disagree with those of relatives. However, the data validity is suggested by the fact that the participating nurses came from 23 different nursing homes. On the other hand, it is possible that a positive selection bias occurred, whereby only nursing homes with ethical practices of managing emergency situations participated.

The generalisability of the present results to other countries may be restricted by the limited legal and professional authority of licensed nurses, as well as the particular organisation of EMS in Germany.^3,25^ Further research is needed on additional factors that may influence the quality of the relationships between nurses and relatives.

## Conclusion

Relatives of nursing home residents may represent important sources of support for nurses in emergency situations. In such situations, relatives’ type and degree of involvement may range from procuring medicines from the pharmacy to taking residents to the doctor’s office, accompanying residents during hospital transfers, and supporting nursing staff in their communication with GPs and residents. At the same time, nurses’ interaction with relatives can be a source of ethical conflict. In particular, ethical challenges may arise in situations in which: (1) nurses have to balance the information needs of relatives with their obligation to provide appropriate care to all residents; (2) relatives demand hospitalisation or an immediate visit to the doctor, although this is not deemed necessary by nurses; (3) relatives demand the initiation of lifesaving treatment, although nurses are convinced that this is contrary to the documented or alleged will of the resident; and (4) nurses want to initiate hospitalisation, but relatives disagree with this course of action.
